# The intake of high fat diet with different *trans *fatty acid levels differentially induces oxidative stress and non alcoholic fatty liver disease (NAFLD) in rats

**DOI:** 10.1186/1743-7075-8-65

**Published:** 2011-09-23

**Authors:** Madiha Dhibi, Faten Brahmi, Amira Mnari, Zohra Houas, Issam Chargui, Linda Bchir, Noureddine Gazzah, Mohammed A Alsaif, Mohamed Hammami

**Affiliations:** 1 Laboratory of Biochemistry, UR: "Human Nutrition and Metabolic Disorder" Faculty of Medicine of Monastir 5019, Tunisia; 2 Laboratory of Histology Cytology and Genetics, Faculty of Medicine, Monastir 5019, Tunisia; 3 College of Applied Medical Sciences, VPP Unit, King Saud University, Riyadh, Saudi Arabia

**Keywords:** *trans *fatty acids, oxidative stress, non alcoholic fatty liver disease, rats

## Abstract

**Background:**

*Trans*-fatty acids (TFA) are known as a risk factor for coronary artery diseases, insulin resistance and obesity accompanied by systemic inflammation, the features of metabolic syndrome. Little is known about the effects on the liver induced by lipids and also few studies are focused on the effect of foods rich in TFAs on hepatic functions and oxidative stress. This study investigates whether high-fat diets with different TFA levels induce oxidative stress and liver dysfunction in rats.

**Methods:**

Male Wistar rats were divided randomly into four groups (n = 12/group): C receiving standard-chow; Experimental groups that were fed high-fat diet included 20% fresh soybean oil diet (FSO), 20% oxidized soybean oil diet (OSO) and 20% margarine diet (MG). Each group was kept on the treatment for 4 weeks.

**Results:**

A liver damage was observed in rats fed with high-fat diet via increase of liver lipid peroxidation and decreased hepatic antioxidant enzyme activities (superoxide dismutase, catalase and glutathione peroxidase). The intake of oxidized oil led to higher levels of lipid peroxidation and a lower concentration of plasma antioxidants in comparison to rats fed with FSO. The higher inflammatory response in the liver was induced by MG diet. Liver histopathology from OSO and MG groups showed respectively moderate to severe cytoplasm vacuolation, hypatocyte hypertrophy, hepatocyte ballooning, and necroinflammation.

**Conclusion:**

It seems that a strong relationship exists between the consumption of TFA in the oxidized oils and lipid peroxidation and non alcoholic fatty liver disease (NAFLD). The extent of the peroxidative events in liver was also different depending on the fat source suggesting that feeding margarine with higher TFA levels may represent a direct source of oxidative stress for the organism. The present study provides evidence for a direct effect of TFA on NAFLD.

## Background

Various food processing techniques have been found to leave deleterious effects on the processed foods and fats and oils are no exception [1-3]. In the developing nations, the intermittent use of reprocessed thermoxidised oil is widespread [4]. Due to their long shelf life, their suitability during deep-frying and their semisolidity, partially hydrogenated vegetable oils are used by the food industries to enhance the palatability of baked goods and sweets. In the process of hydrogenation, unsaturated vegetable oils undergo the introduction of hydrogen gas under certain conditions of pressure and temperature using a catalyst metal (nickel, palladium, platinum, and ruthenium). The hydrogenation process involves the transformation of certain unsaturated fatty acids from *cis *to *trans *configuration. In their natural form, most fatty acids present only *cis-*isomerism [5]. T*rans *fatty acids (TFAs) are produced through the industrial hardening of the vegetable oils to make the products more stable and robust, and thus easier to handle or store [6]. Most TFA have physical properties similar to saturated fatty acids (SFA) [7]. More specifically, monounsaturated TFA isomers with 18-carbon chain length (*trans*-18:1) are some of the predominant TFAs present in the human diet [8,9]. TFAs are known as a risk factor for coronary vascular diseases (CVD), insulin resistance and obesity accompanied by systemic inflammation, the features of metabolic syndrome [10,11]. Recent studies suggest multiple possible mechanisms that might mediate the association of TFAs with CVD [12]. For example, TFAs influence prostaglandins balance, which in turn promotes thrombogenesis [13] and inhibits the conversion of linoleic acid to arachidonic acid and to other n-6 PUFA, perturbing essential fatty acid metabolism and causing changes in the phospholipid fatty acid composition in the aorta [14]. TFAs have been associated with the activation of systemic inflammatory responses, including substantially increased levels of IL-6, TNF-α, TNF receptors and monocyte chemoattractant protein-1 [15]. Furthermore, TFAs have been associated with increased levels of several markers of endothelial activation, including soluble intercellular adhesion molecule 1, soluble vascular-cell adhesion molecule 1 and E-selectin [10]. TFAs are postulated to be involved in promoting vascular dysfunction, as reflected by a reduction in brachial artery flow [16]. These observations suggest that TFAs are linked to the development of CVD, probably via a vascular pro-inflammatory response [17]. Oxidative damage is a major contributor to the development of CVD. Nevertheless, little is known about the effects on the liver induced by lipids [6] and few studies are focused on the effect of foods rich in TFAs on hepatic functions and oxidative stress. Oxidative stress results from an imbalance between oxidant production and antioxidant defenses [18]. Oxidative stress induced by free radicals has been linked to the development of several diseases such as cardiovascular, cancer, and neurodegenerative diseases [19]. When cellular antioxidant mechanisms are overwhelmed, a long-term decline in their antioxidant capacity causes the oxidative stress [20,21]. Oxidative stress is now believed to be an important factor in the development of non alcoholic fatty liver disease (NAFLD) [20,22]. NAFLD is the most common liver disorder in the world, and in obesity, type 2 diabetes and related metabolic diseases, its incidence reaches 70-90% [23]. The disease is characterized by the accumulation of triacylglycerols inside liver cells, and the condition can progress into more serious liver disease, such as non alcoholic steatohepatitis, liver fibrosis, cirrhosis, and more rarely, liver carcinoma [23]. Previous works have shown that feeding rats a high fat diet (57% of energy from fat) induces hepatic steatosis and liver damage, which are characteristic of NAFLD and thus provides a suitable model for the early stages of the disease [24,25]. But, in these studies TFAs in the fat diet were not investigated and neglected. Therefore, it is necessary to examine the relationship between the liver functions and TFAs consumption in dietary lipids.

We investigated whether high-fat diet (fresh soybean oil, oxidized soybean oil and margarine) with different TFA levels induces oxidative stress and NAFLD in rats.

## Materials and methods

### Analytical determinations of supplemented dietary fat

Soybean oil and margarine were purchased in a local supermarket. The thermoxidized oil was prepared by heating soybean oil in an oven set for 24 hours at 200°C. The extent of lipid peroxidation was determined by assaying the peroxide value and UV absorbance at 232 and 270 nm (k232 and k270) and p-anisidine value according to the European Official Methods (EEC 2568/91) [26]. the oxidative stability index (OSI) was evaluated by the Rancimat apparatus (Mod. 743, Metrohm Ω, Switzerland) using an oil of 3 g warmed to 120°C and an air flow of 20 L/h [27]. Results were expressed as induction time in hours of hydroperoxides decomposition.

### Determination of fatty acid profile

Fatty acid methyl esters (FAMEs) from the oil samples were prepared as described by Issaoui et al. [28]. Individual FAMEs were separated and quantified by gas chromatography using a Model 5890 Series II instrument (Hewlett-Packard, Palo Alto, CA) equipped with a flame ionisation detector, and a fused silica capillary column DB-23 (60 m length, 0.32 mm i.d., and 0.25 μm film thickness; HP-Agilent Technologies, Wilmington).

### Determination of antiradical activity

The capacity to scavenge the "stable" free radical 2,2-dipheny1-1-picrylhydrazyl (DPPH) was monitored according to the method of Ramadan and Morsel [29]. The solution was incubated at room temperature for 60 min and the decrease in absorbance at 515 nm was determined after 1, 30 and 60 min using a UV-visible spectrophotometer (Perkin Elmer Lambda 25).

### Animal treatment

Male adult Wistar rats (Central Pharmacy, Tunisia), weighing about 200 to 280 g, were housed at 22 ± 3°C, with 12- hour light-dark periods, a 40% minimum relative humidity and free access to water and standard diet: protein 17% (methionine and choline accounting 3000 and 2720 milligrams per kilogram, respectively), carbohydrate 62%, lipids 4%, ash 7%, and moisture 10% (SICO, Sfax, Tunisia). All the breeding phases and experiments were conformable to the rules of the Tunisian Society for the Care and Use of Laboratory Animals. All experiments were conducted at the animal facilities of the faculty of Medicine, Monastir; with the approval of the Faculty of Medicine Ethics committee. After acclimatization to the laboratory conditions for one week, the animals were divided into 4 groups of 12 animals each. Group C included the control animals and received standard chow. Experimental groups that were fed high-fat diet included 20% fresh soybean oil diet (FSO), 20% oxidized soybean oil diet (OSO) and 20% margarine diet (MG). Each group was kept on the treatment for 4 weeks. Water and food consumption and the individual animal body-weight were recorded daily throughout the experiment. At the end of the experimental period, the rats were kept fasting overnight and were sacrificed under diethyl ether anesthesia.

### Biochemical analysis of liver functions

Serum Alkaline Phosphatase (ALP) Aspartate Transaminase (AST), Alanine Transaminase (ALT) and Lactate Dehydrogenase (LDH) activities were determined spectrophotometrically using commercial diagnostics kits supplied by Randox Laboratories (Ardmore, Northern Ireland, UK).

### Measurement of TBARS levels

According to Buege and Aust [30], lipid peroxidation was estimated by measuring thiobarbituric acid reactive substances (TBARS) and expressed in terms of malondialdehyde (MDA) content. For the assay,125 μl of supernatant (S1) were mixed with 50 μl of saline buffer (PBS, PH 7.4),125 μl of 20% trichloroacetic acid containing1% butylhydroxytoluene and centrifuged (1000 g, 10 min,4°C). Then, 200 μl of supernatant (S2) was mixed with 40 μl of HCl (0.6M) and 160 μl of Tris-thiobarbituric acid (120 mM) and the mixture was heated at 80°C for 10 min. The absorbance was measured at 530 nm. The amount of TBARS was calculated using an extinction coefficient of 1.56 × 10-^5 ^M^-1 ^cm-^1 ^and expressed in nmol of MDA/mg protein.

### Measurement of conjugated dienes

Conjugated dienes were determined by the method of Recknagel and Ghoshal [31]. A portion of tissue homogenate was transferred to a chloroform/methanol mixture (2:1). The whole mixture was vortexed and centrifuged at 2500 g. The upper layer was washed with chloroform/methanol/H_2_O and centrifuged. The lower layer was combined with the first lower layer and evaporated under N_2_. The extract was redissolved in 1 ml cyclohexane. Absorbance was determined at 233 nm. An extinction coefficient of 2.52 × 10^4 ^mole^-1 ^was used. Results were expressed as mmoles mg-1 protein.

### Liver antioxidant enzymes activities

Superoxide dismutase (SOD) activity in liver homogenate was assayed spectrophotometrically as described by Beyer and Fridovich [32]. This method is based on the capacity of SOD to inhibit the oxidation of nitroblue tetrazolium (NBT). One unit of SOD represents the amount of enzymes required to inhibit the rate of NBT oxidation by 50% at 25°C. The activity was expressed as units/mg protein.

Catalase (CAT) activity was measured at 20°C by a slightly modified version of Aebi's method [33]. Hydrogen peroxide (H_2_O_2_) decomposition by CAT enzyme was monitored kinetically at 240 nm. The molar extinction coefficient of 0.043 mM^-1^cm^-1 ^was used to determine CAT activity. One unit of activity is equal to the micromole of H_2_O_2 _degraded per minute per milligram of protein.

Glutathione peroxidase activity (GPx) was assayed according to the method of Flohe and Gunzler [34]. The activity was expressed as mmol of GSH oxidized/min/mg of protein at 25°C.

### Protein assay

Protein concentrations in the liver were determined according to the method of Bradford [35] using bovine serum albumin as a standard.

### Statistical analysis

The data were analyzed using the Statistical Package for Social Sciences (SPSS) program, release 11.0 for Windows (SPSS, Chicago, IL, USA). In each assay, the experimental data represent the mean of 12 independent assays ± standard deviations. Duncan's test was used to determine any significant differences between different groups. The statistical significance was set at *p *< 0.05. The results were analyzed using the Student t test for comparison between the dietary fat parameters. To point out the correlation between the analyzed parameters, Pearson's test was carried out.

## Results and discussion

### Analytical parameters of the dietary fat

The analytical parameters of the dietary fat employed are shown in Table 1. It is very important to assess the oxidative degradation of fats and oils, because free-radical initiated oxidation is one of the main causes of rancidity in fats and oils, which results in the alteration of major quality control variables such as color, flavor, aroma and nutritional value [36]. The thermally oxidized soybean oil (OSO) samples composition were different from the fresh soybean oil (FSO) with a high peroxide, conjugated dienes and p-anisidine value (Table 1) and a significant reduction of oxidative stability (3.74 *vs*. 0.67 h) and antiradical capacity (93.12 vs. 55.16%), respectively (*p *< 0.01). Margarine (MG) samples also showed higher antioxidant ability (77.9%) and oxidative stability index (4.27 h) and a lower *p*-anisidine and extinction coefficient value than OSO (Table 1).

**Table 1:** Mean values of fatty acid composition (%), lipid peroxidation parameters and antiradical properties of high-fat diet (fresh soybean oil, FSO; oxidized soybean oil, OSO and margarine, MG)

	Supplemented high-dietary fat		
	
	FSO	OSO	MG
**Fatty acids (%)**			
8:0	nd	nd	0.26 ± 0.00^##^
10:0	nd	nd	0.26 ± 0.00^##^
12:0	nd	nd	3.16 ± 0.01^##^
14:0	0.08 ± 0.002	0.08 ± 0.01	1.84 ± 0.03^##^
14:1	0.02 ± 0.00	0.02 ± 0.009	0.04 ± 0.00^#^
16:0	10.96 ± 0.06	12.08 ± 0.01**	30.33 ± 0.04^##^
***trans- *16:1 n-7**	**0.02 ± 0.00**	**0.024 ± 0.006**	**0.03 ± 0.00^#^**
*cis-*16:1 n-7	0.09 ± 0.00	0.11 ± 0.00**	0.13 ± 0.00^#^
17:0	0.29 ± 0.02	0.28 ± 0.01	0.20 ± 0.01^##^
17:1	0.08 ± 0.02	0.08 ± 0.00	0.05 ± 0.01^##^
18:0	4.82 ± 0.04	3.93 ± 0.01**	4.8 ± 0.01^##^
***trans-*18:1 n-9**	**nd**	0.117 ± 0.01**	**1.78 ± 0.13^##^**
*trans-*18:1 n-7	nd	nd	nd
*cis-*18:1 n-9	21.96 ± 0.2	25.22 ± 0.02**	30.1 ± 0.13^##^
*cis-*18:1 n-7	1.29 ± 0.05	1.71 ± 0.01**	0.73 ± 0.00^##^
**18:2 n-6 (t9. t12)**	**0.07 ± 0.00**	0.138 ± 0.01**	**0.096 ± 0.001^#^**
**18:2 n-6 (t9. c12)**	**nd**	0.054 ± 0.003**	**0.052 ± 0.004**
**18:2 n-6 (c9. t12)**	**0.09 ± 0.01**	0.288 ± 0.1**	**0.2 ± 0.01^##^**
18:2 n-6 (c9. c12)	50.75 ± 0.04	48.12 ± 0.01**	21.73 ± 0.3^##^
*cis-*18:3 n-6	0.19 ± 0.00	0.40 ± 0.05**	0.14 ± 0.00^##^
***trans***-**18:3 n-3**	**0.02 ± 0.00**	0.366 ± 0.01**	**0.016 ± 0.011^##^**
*cis-*18:3 n-3	7.65 ± 0.1	4.76 ± 0.02**	2.55 ± 0.02^##^
**18:2 (c9. t11)**	**0.024 ± 0.001**	0.099 ± 0.003**	**0.068 ± 0.00^##^**
**18:2 (t10. c12)**	**0.013 ± 0.001**	0.056 ± 0.004**	**0.042 ± 0.00^#^**
20:0	0.43 ± 0.01	0.43 ± 0.002	0.35 ± 0.00^##^
***trans-*20:1 n-9**	**0.026 ± 0.00**	0.199 ± 0.006**	**0.06 ± 0.00^##^**
20:1 n-9	0.24 ± 0.01	0.2 ± 0.080*	0.18 ± 0.02^#^
20:2 n-9	0.08 ± 0.003	0.09 ± 0.002	0.02 ± 0.00^##^
20:3 n-6	0.03 ± 0.00	0.04 ± 0.02**	0.02 ± 0.00
20:4 n-6	0.03 ± 0.00	0.03 ± 0.00	0.01 ± 0.00^#^
22:0	0.06 ± 0.00	0.067 ± 0.00*	0.03 ± 0.00^##^
Σ**SFA**	**16.22 ± 0.13**	16.9 ± 0.03**	**41.42 ± 0.1^##^**
Σ**cis MUFA**	**23.6 ± 0.3**	27.47 ± 0.1**	**31.25 ± 0.13^##^**
Σ **cis PUFA**	**59.36 ± 0.12**	54.21 ± 0.1**	**24.81 ± 0.03^##^**
**Total TFAs**	**0.226 < 1**	**1 < 1.23 < 2**	**2.4 > 2**
**Lipid peroxidation**			
Oxidative stability index (h)	3.74 ± 0.01	0.67 ± 0.04**	4.27 ± 0.63^##^
Peroxide value (meq O_2_/kg)	2.66 ± 0.00	6 ± 0.00**	17.33 ± 0.94^##^
p-anisidine value	2.13 ± 0.7	7.5 ± 2.2**	2.61 ± .024^##^
k232 (conjugated dienes)	2.77 ± 0.10	4.26 ± 0.04**	3.9 ± 0.07
k270	1.11 ± 0.05	4.01 ± 0.05**	2.54 ± 0.15^##^
**Antiradical ability: **DPPH (%)	93.12 ± 0.06	50.16 ± 2.88**	77.91 ± 0.5^##^

nd: not detected.

Values are given as mean ± SD (n = 3). **FSO**: Fresh soybean oil, **OSO**: oxidized soybean oil,

**MG**: margarine.

*p < .05, OSO *vs*. FSO; **p < .01 OSO *vs*. FSO. **^#^***p *< .05, MG *vs*. OSO; **^##^***p *< .05 MG *vs*. OSO. Comparison between supplemented-diet compositions was made using unpaired Student t test.

Concerning the fatty acid (FA) composition, as shown in Table 1, FSO and OSO were characterized by the presence of high levels of polyunsaturated fatty acid (PUFA) fraction with a significant (*p *< 0.01) difference (59.36 vs. 54.21% respectively). Whereas, MG was distinguished by the presence of SFA (41.42%) and a significant low level of PUFA (24.81%). For TFA isomers, FSO contained about 0.22% of total FA (Table 1). Detection of TFA isomers in FSO confirms the fact that the oil retailed in the market even without thermal treatment has already started deteriorating. This also could be due to the refining process effect. MG samples contained higher amounts of total TFAs accounting 10 and 1.23 times than FSO and OSO, respectively. As reported by Assumpção et al. [37], during hydrogenation, the double bonds of FA that form triacylglycerols change their position and produce *trans*-geometric isomers. In MG samples, the *trans *18:1 n-9 constituted the highest proportion among the identified *trans*-isomers, whereas polyunsaturated *trans*-isomers appeared only in small quantities. However, for OSO, *trans *PUFA represent about 60% of total TFA. This is in accordance with Mayneris-Perxachs et al. [38] who reported that the predominant *trans *isomers in industrially processed products is elaidic acid (*trans*-9 C18:1) and in agreement with Lichtenstein [39] who reported that the majority of TFAs in the diet are *trans*-18: 1, which is derived from the partial hydrogenation of oils. However, the process of heating vegetable oils during deodorization and frying or baking food in vegetable oils results in the generation of *trans*-18:2 [40].

**Figure 1:**
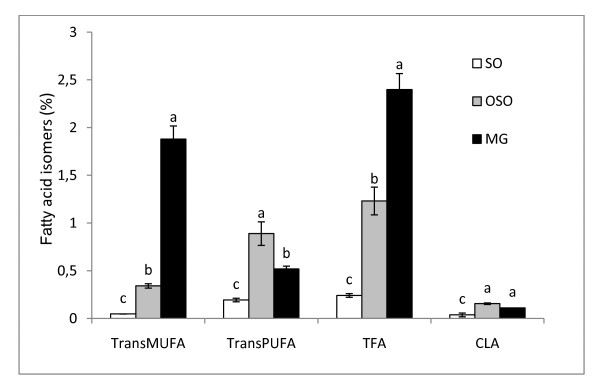
**Fatty acid isomers in dietary fat diet**. *trans *MUFA: *trans *monounsaturated fatty acid, *trans *PUFA: *trans *polyunsaturated fatty acid, TFA: total *trans *fatty acid, CLA: conjugated linoleic acid. Data are expressed as means ± SD (n = 3). Comparison between groups was made using Duncan's test. Values followed by different subscript letters are significantly different. (*p *< 0.05).

Thus, Supplemented dietary fat contained different levels of total TFAs ranged from proportions of total fat <1%, <2% and > 2% in FSO, OSO and MG diets respectively (Figure 1). In addition, the isomer type also differs with predominance of *trans*-18:2 in oils and *trans*-18: 1 n-9 in MG. In observational studies utilizing biomarkers of TFAs consumption, both 18:1 and 18:2 isomers appear to contribute to risk of CVD [41].

### Growth and nutritional status of rats

Most rats gained weight consistently during the four-week dietary treatments. The average body weight gain at the end of the four weeks was 16.4 g in the control animals, 24.55, 20.33 and 25.83 g in FSO-fed, OSO-fed and MG-fed rats, respectively (Table 2). Following four weeks of feeding, the body weight gained in high-fat fed rats was statistically not significant (Table 2). The feeding efficiency of rats fed with the OSO diet was lower than that of the FSO-fed rats. This may be explained by the fact that almost all amino acids react with primary and secondary products of oxidized lipids, thereby decreasing the digestive utilization of protein, amino acids and fats, which may affect a weight gain [42]. On the other hand, results showed that FSO diet significantly increased the absolute liver weight compared with the control group. However, no significant changes were observed for OSO and MG groups (Table 2).

**Table 2:** Body weight gain, food intake, water intake and feed efficiency of rats fed with fresh soybean oil (FSO) oxidized soybean oil (OSO) and margarine (MG).

Growth and nutritional status of rats					
	**Body weight gain (g)**	**Liver weight (g)**	**Food intake (g/day)**	**Water intake (ml/day)**	**Feed efficiency (B.W gain/food intake)**
	
***C***	16.4 ± 6.67^a^	6.92 ± 1.42 ^a^	16.03 ± 2.47^a^	9.33 ± 1.91 ^ab^	1.02
***FSO***	24.55 ± 9.7 ^a^	8.15 ± 0.83 ^b^	14.94 ± 1.82 ^ab^	10.04 ± 1.53 ^a^	1.64
***OSO***	20.33 ± 7.81 ^a^	7.54 ± 0.64 ^ab^	13.74 ± 2.41^c^	9.82 ± 1.5 ^ab^	1.47
***MG***	25.83 ± 6.64 ^a^	7.27 ± 0.7 ^a^	14.24 ± 2.17 ^bc^	9.07 ± 1.22 ^b^	1.81

Data are expressed as means ± SD (n = 12 rats per group). Control group: **C**; Fresh soybean-oil fed group: **FSO; **oxidized soybean oil-fed group: **OSO**; margarine-fed group: **MG**. Comparison between groups was made using Duncan's test. Different parameters values followed by different subscript letters (a, b and c) are significantly different between groups. (*p *< 0.05).

### Biochemical indicators of liver function

The levels of plasma hepato-specific enzymes such as, ALP and LDH were significantly increased (*p *< 0.05) in high-fat fed rats compared to control (Table 3). Feeding (OSO) led to significant higher levels of AST, ALP and LDH in comparison to fresh oil fed group (*p *< 0.05). Enhanced levels of plasma ALT and AST are indicative of liver damage [43]. Plasma ALP is a sensitive detector for intrahepatic and extrahepatic bile obstruction [44]. It is well known that dietary fat sources strongly influence several biochemical variables both in plasma and in biological membranes [45-47]. Consumption of OSO and MG diets causes a significant increase of biochemical indicators of liver damage. We noticed a close positive correlation between TFA levels in dietary fat and AST, ALAT, ALP and LDH (Table 4). These results revealed hepatic damage in rats consumed TFA.

**Table 3:** Biochemical indicators of liver function in plasma in control (C) and high fat treated rats fed a diet with fresh soybean oil (FSO), oxidized soybean oil (OSO) and margarine (MG)

Plasma hepato specific enzymes (U/L)				
	**AST**	**ALT**	**ALP**	**LDH**
**C**	120.5 ± 36.06^a^	55.25 ± 4.03 ^a^	167.85 ± 28.8^a^	410 ± 20 ^a^
**FSO**	145.5 ± 2.38 ^ab^	58.5 ± 8.3 ^a^	217.71 ± 36.9 ^b^	585.5 ± 87.1 ^b^
**OSO**	162.8 ± 15.12 ^b^	61 ± 9.02 ^a^	269.33 ± 10.21 ^c^*	860.5 ± 13.43^c^**
**MG**	207 ± 7.3 ^c++^	76.83 ± 9.23 ^b+^	248.5 ± 13.7 ^bc^	981.5 ± 118.4 ^c^

Data are expressed as means ± SD (n = 12 rats per group). **C**: controls group, **FSO**: Fresh soybean oil fed group, **OSO**: oxidized soybean oil fed group, **MG**: margarine fed group. Alkaline Phosphatase: ALP; Aspartate Transaminase: AST; Alanine Transaminase: ALT; lactate dehydrogenase: LDH

Comparison between groups was made using Duncan's test. Different parameters values followed by different subscript letters (a, b and c) are significantly different between groups. (*p *< 0.05).

*p < .05, OSO *vs*. FSO group; **p < .01, OSO *vs*. FSO group. ^+^*p *< .05, MG vs OSO group; ^++^*p *< .05, MG *vs*. OSO group. Comparison between groups was made using unpaired Student test.

**Table 4:** Correlation between fatty acid isomers in the diet and oxidative stress parameters in rat's liver and plasma hepato-specific enzymes

	SOD	*CAT*	*GPx*	*CD*	*MDA*	*AST*	*ALT*	*PAL*	*LDH*
***trans MUFA***	-0.977	-0.952	-0.770	0.105	0.626	0.992	1.000*	0.258	0.829
***trans PUFA***	-0.321	-0.409	-0.719	1.000**	0.844	0.235	0.087	0.989	0.649
**total TFAs**	-0.994	-1.000*	-0.934	0.418	0.843	0.980	0.939	0.554	0.964

*p < .05; **p < .01

MUFA: monounsaturated fatty acid; PUFA: polyunsaturated fatty acid; TFAs: *trans *fatty acids; alkaline phosphatase: ALP; aspartate transaminase: AST; alanine transaminase: ALT; lactate dehydrogenase: LDH; SOD: superoxide dismutase; GPx: glutathione peroxidase; CAT: catalase; CD: conjugated dienes; MDA: malondialdehyde.

### Liver's Lipid peroxidation

When compared to control group, we found a clear evidence of liver's lipid peroxidation of FSO, OSO and MG-fed rats, as judged by their significantly high content of conjugated dienes (CD) products, reflecting the initial phase of lipid peroxidation.

**Figure 2:**
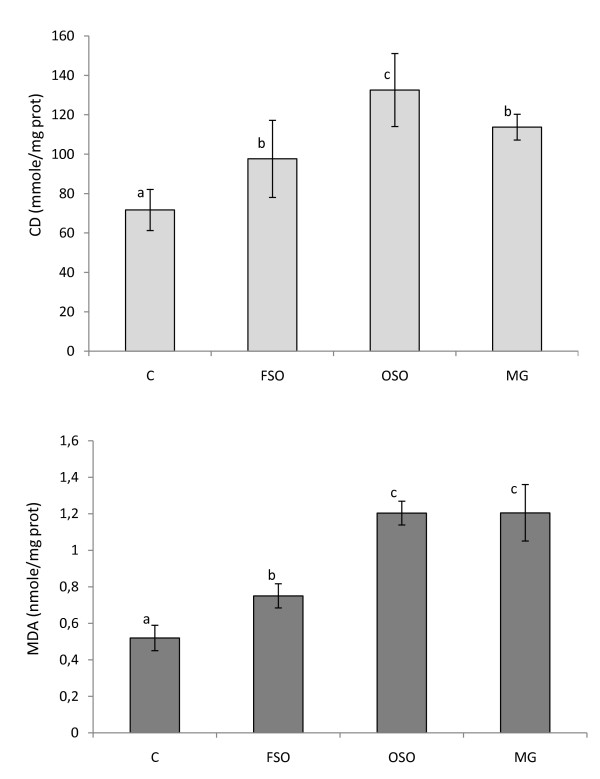
**Malondialdehyde (MDA) and conjugated dienes (CD) in the liver of rats fed with high fat diet with different *trans *fatty acid levels**. C: controls group, FSO: Fresh soybean oil fed group, OSO: oxidized soybean oil-fed group, MG: margarine-fed group. Data are expressed as means ± SD (n = 12 rats per group). Comparison between groups was made using Duncan's test. Values followed by different subscript letters are significantly different. (*p *< 0.05).

On the other hand, when the degradative phase of lipid peroxidation was examined, assaying thiobarbituric acid reacting substances (TBARS), the MDA levels in the FSO group, comparing to the C group, was increased by 26.5% (Figure 2). The TBARS in the livers of high-fat fed animals were found to be significantly increased compared to control rats (*p *< 0.05). Elevated levels of TBARS in liver are a clear manifestation of excessive formation of free radical and activation of lipid peroxidation.

Our findings revealed that the rates of hepatic lipid peroxidation were markedly higher in margarine and OSO-fed groups than in the fresh oil fed group. However, for OSO-fed group, the CD concentration was significantly increased by 85% and 36% of that in C and FSO group respectively. The results from lipid peroxidation measurements confirm that the loss of antioxidant capacity and the increase of TFAs in OSO affect the liver function, suggesting that feeding oxidized oil may represent a direct source of oxidative stress for the organism. A positive correlation between the level of total TFAs in the diet and the concentration of the TBARS in the liver of high-fat fed animals (r = 0.84) was observed. A highly significant positive correlation was also noted between CD levels in rat's liver and *trans *PUFA in the rat diet (r = 1.0; *p *< 0.01) (Table 4). The importance of FAs resides in the finding that biological membranes adapt their composition according to that of dietary fat [48-50]. Dietary FAs can influence the susceptibility of cells to oxidative stress, perhaps due to changes in cell membrane FA composition [51]. As well known, lipid peroxidation starts with abstraction of OH• from a -CH2- group of PUFA, where the carbon radical is usually stabilized by a molecular rearrangement forming conjugated dienes, compounds containing two double bonds separated by a single bond. Conjugated dienes react with O_2 _forming peroxyl radicals that react with OH• atoms from other lipids, producing lipid hydroperoxides or forming cyclic peroxides, and several products are formed, including MDA [52]. Lipid peroxidation is the process of oxidative degradation of PUFAs and its occurrence in biological membranes causes impaired membrane function, structural integrity, decrease in membrane fluidity and inactivation of a several membrane bound enzymes [53]. Niu *et al*. have reported that phospholipids in biological membranes containing TFAs are known to attract cholesterol [54]. This phenomenon plausibly alters cell membrane structure, including redefining lipid raft and non-raft regions in size, organization and composition. Lipid rafts are important for cellular signalling, as they provide docking sites for receptors, co-receptors and mediators including adhesion molecules [55]. Recent animal experiments indicate that TFAs impair fat cell membrane fluidity. When TFAs are incorporated into cell membranes, the membrane fluidity is reduced and the cells do not function as well. The resulting effect is then to promote further production of reactive oxygen species which explain the increase in lipid peroxidation in groups fed with TFAs diet.

### Liver's activities of antioxidant enzymes

**Figure 3:**
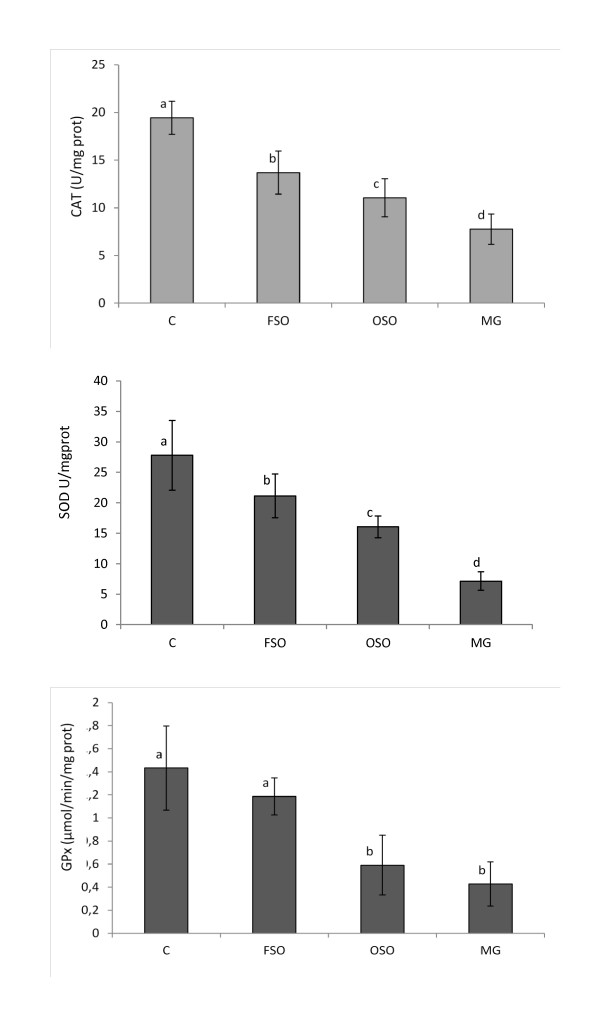
**Antioxidant enzyme activities in the liver of rats fed with high fat diet with different *trans *fatty acid levels**. CAT, SOD and GPx of rat's liver exposed to different high-fat diets. C: controls group, FSO: Fresh soybean oil-fed group, OSO: oxidized soybean oil-fed group, MG: margarine-fed group. Data are expressed as means ± SD (n = 12 rats per group). Comparison between groups was made using Duncan's test. Values followed by different subscript letters are significantly different. (*p *< 0.05).

The removal of reactive oxygen substances is accomplished by enzymatic and non-enzymatic reactions in biological systems. In enzymatic reactions, SOD converts superoxide anions to hydrogen peroxide (H_2_O_2_), and H_2_O_2 _can be rapidly degraded by CAT and GPx to H_2_O [56]. The activities of SOD and CAT in the liver were significantly (*p *< 0.05) lowered in rats fed with high-fat diet than control group animals (Figure 3). Loss of CAT activity results in oxygen intolerance and triggers a number of deleterious reactions such as protein and DNA oxidation, and cell death [52]. The GPx activity was significantly decreased in liver of rats fed with OSO and MG diet as compared to the control and FSO-fed rats (*p *< 0.05) (Figure 3). High-fat diets can cause the formation of toxic intermediates that can inhibit the activity of antioxidant enzymes [57] and the accumulation of O_2_^- ^radicals and H_2_O_2 _which in turn forms hydroxyl radicals [58]. The activities of SOD and CAT were significantly decreased in OSO group than FSO group (*p *< 0.05) (Figure 3). A close negative correlation was noted between TFA levels in the diet and SOD (r = -0.99), CAT(r = -1.0) and GPx (r = -0.93) activities in rat's liver suggesting that increasing consumption of TFAs is associated with the decrease of the efficiency of the antioxidant-enzymatic system and therefore, with the increase of oxidative stress in rat's liver. TFAs may impart their effect by enhancing intrinsic signaling mechanisms leading to a chronic, pro-inflammatory state. Consumption of diets high in TFAs may induce long-term progressive changes in the antioxidant enzyme's activities.

### Histopathological lesions

**Figure 4:**
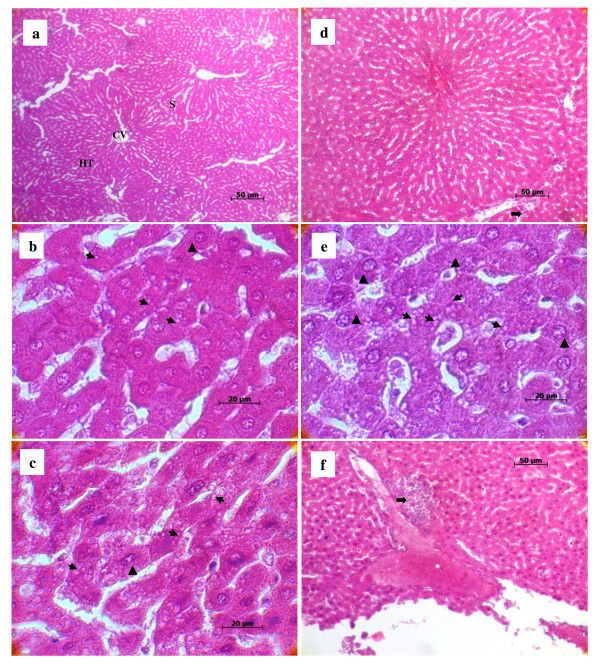
**Effect of high fat diet with different *trans *fatty acid levels on rat's liver histology**. Normal liver histological aspect from a control (H&E 32 ×). Panel (a) it is composed of hexagonal or pentagonal lobules with central veins (CV) and peripheral hepatic triads (HT) embedded in connective tissue. Hepatocytes are arranged in trabecules running radiantly from the central vein and are separated by sinusoids (S) containing Kuppfer cells. Liver from experimental groups (H&E 100×): FSO Panel (b): moderate lipid accumulation is seen in many hepatocytes; OSO Panel (b); abundance of cytoplasm vacuolization and ballooned hepatocytes and MG Panel (e); severe lipid accumulation in hepatocytes and high number of ballooned hepatocytes. Cytoplasm vacuolization in parenchymatous cells of the liver (thin arrow), hypertrophied hepatocytes (black triangle). Liver from OSO Panel (d) and MG Panel (f) groups (H&E 32×): photomicrograph of degenerated hepatocytes and necrosis (thick arrow).

Histopathologically, liver sections from rats fed with the standard diet had shown normal morphological appearance (Figure 4a). Livers of the experimental groups showed a clear difference from those of the control group. In the group that fed FSO, the initial phase of NALFD, during which fat accumulates in the liver (Figure 4b, thin arrow). and cytoplasm vacuolation of hepatocytes were observed (Figure 4b, black triangle). As previously reported by Samuhasaneeto *et al*. [59], one hundred percent fat diet caused mobilizing of free fatty acid from adipose tissue and transporting into hepatocytes. These results are in agreement with previous studies of the effects of high-fat diet in inducing the early stage of NAFLD [59].

Feeding OSO for four weeks, rat's liver showed increased incidences of hepatocytes hypertrophy (Figure 4c, black triangle), fat deposition (Figure 4c, thin arrow) and infiltration of a mixed population of inflammatory cells in the liver, as well as ballooning degeneration of hepatocytes characterized by cell swelling with empty intracellular content, indicating cell necrosis (Figure 4d, thick arrow). As known, dietary lipids in the form of chylomicrons are transported from the gut via the lymphatic system to the liver where they are incorporated after release from lipoproteins by hepatic lipoprotein lipase [60]. Physiologically and during the postprandial phase, dietary lipids are stored in the liver, where they are processed and assembled with apolipoprotein B 100 (ApoB) to form very-low-density lipoprotein (VLDL). These particles are secreted and distribute lipids to lipid-storing adipose tissue [60]. When the hepatocyte is injured, plasma membrane can be disrupted and the leakage through extra-cellular fluid of the enzyme occurs where they can be detected at abnormal levels in the serum [61]. This is clearly evident by a substantial increase in plasma levels of AST, ALP and LDH in OSO group (Table 3). Previous studies have reported that *trans *fats appear to affect lipid metabolism through several pathways. In vitro, TFAs alter the secretion, lipid composition, and size of apolipoprotein B-100 (apoB-100) particles produced by hepatic cells [62,63]. The liver failed to synthesize apolipoprotein that was used for packaging and exporting of fat from the liver. Therefore, triglycerides accumulated in the liver [64]. As reported by Mensink et al [65], *trans *fats increase the blood levels of triglycerides as compared with the intake of other fats. In this study, triglycerides levels were found to be increased in the plasma of rats fed with MG diet followed by OSO diet and FSO diet (data not published). The higher inflammatory response in the liver was induced by MG diet. Liver histopathology from MG group showed severe cytoplasm vacuolation, hepatocyte hypertrophy **(**Figure 4e, black triangle) and a noticeable hepatocyte ballooning demonstrating a large area of necroinflammation (Figure 4f, thick arrow). The histological and pathogenic features of NAFLD were clearly developed in the MG group which is submitted to margarine diet with TFA level reaching the 2% of total fat. Previous studies proved that oxidative stress is now believed to be an important factor in the development of NALFD [66]. These alterations in the liver of rats fed with OSO and MG diet containing respectively more than 1% and 2% TFAs of total fat implicate TFAs in triggering the development of NAFLD and/or accelerating the progression of the disease.

## Conclusion

In conclusion, oxidized edible oils fed to rats for four weeks induced lipid peroxidation in liver compared with the same non-oxidized oils. It seems that a strong relationship exists between the consumption of TFAs in the oxidized oils and lipid peroxidation. The extent of the peroxidative events in liver was also different depending on the fat source suggesting that feeding margarine with higher TFA level may represent a direct source of oxidative stress for the organism. The present study provides evidence for a direct effect of TFAs on liver dysfunction causing the disturbances in liver lipid metabolism that result in NAFLD which is a key component of the cardiometabolic syndrome. This suggests that TFAs may influence risk factors for CVD.

## List of Abbreviations

SFA: saturated fatty acid; MUFA: monounsaturated fatty acid; PUFA: polyunsaturated fatty acid; TFAs: trans fatty acids; HDL: high density lipoprotein; LDL: low density lipoprotein; ALP: alkaline phosphatase; AST: aspartate transaminase; ALT: alanine transaminase; LDH: lactate dehydrogenase; SOD: superoxide dismutase; GPx: glutathione peroxidase; GR: glutathione reductase; CAT: catalase; CD: conjugated dienes; MDA: malondialdehyde; CVD: cardiovascular disease; NAFLD: non alcoholic fatty liver disease.

## Competing interests

The authors declare that they have no competing interests.

## Authors' contributions

MD carried out the studies, acquired the data, performed the data analysis, drafted and revised the manuscript. FB and AM played a major role in all the experimental procedures of this study. ZH carried out histological observations. IC was involved in the experimental work performed towards this manuscript. LB participates in carrying out of dietary fat analysis. NG provided technical assistance in the chromatographic analysis. MA provided academic helping service in performing this study. MH involved in the design and organization of the study, interpreted the results and revised the manuscript. All authors have read and approved the final manuscript.

## References

[B1] Gurr AI James AI Lipid Biochemistry An introduction 1975 2 England. Wiley Publishers 50 96

[B2] Kubows S Routes of formation and toxic consequences of lipid oxidation products in foods Free Rad Bio Med 1992 12 63 81 10.1016/0891-5849(92)90059-P 1537572

[B3] Ononogbu CI Lipids in Human Existence 2002 Nig.: AP Express Publishers 105 123

[B4] Jimoh FO Odutuga AA Obaleye JA Changes in Oxidized Groundnut Oil and its Effect on Na+/k+ - Atpase in Rat Tissues Pakistan J Nut 2007 6 1 63 67

[B5] Sommerfeld M Trans unsaturated fatty acids in natural products and processed foods Prog Lipid Res 1983 22 221 10.1016/0163-7827(83)90010-3 6356151

[B6] Obara N Fukushima K Ueno Y Wakui Y Kimura O Tamai K Kakazu E Inoue J Kondo Y Ogawa N Sato K Tsuduki T Ishida K Shimosegawa T Possible involvement and the mechanisms of excess trans-fatty acid consumption in severe NAFLD in mice J Hepatol 2010 53 326 334 10.1016/j.jhep.2010.02.029 20462650

[B7] Ohlrogge JB Perkins EG Distribution in human tissues of fatty acid isomers from hydrogenated oils Dietary Fats and Health 1983 Champaign, IL: American Oil Chemist's Society 359 376

[B8] Eckel RH Borra S Lichtenstein AH Yin-Piazza SY Understanding the complexity of *trans *fatty acid reduction in the Americandiet: American Heart Association Trans Fat Conference 2006: report of the Trans Fat Conference Planning Group Circulation 2007 115 2231 2246 10.1161/CIRCULATIONAHA.106.181947 17426064

[B9] van de Vijver LP Kardinaal AF Couet C Aro A Kafatos A Steingrimsdottir L Amorim Cruz JA Moreiras O Becker W van Amelsvoort JM Vidal-Jessel S Salminen I Moschandreas J Sigfusson N Martins I Carbajal A Ytterfors A Poppel G Association between trans fatty acid intake and cardiovascular risk factors in Europe: the TRANSFAIR study Eur J Clin Nutr 2000 54 126 135 10.1038/sj.ejcn.1600906 10694783

[B10] Lopez-Garcia E Schulze MB Meigs JB Manson JE Rifai N Stampfer MJ Consumption of *trans *fatty acids is related to plasma biomarkers of inflammation and endothelial dysfunction J Nutr 2005 135 562 566 15735094 10.1093/jn/135.3.562

[B11] Sun Q Ma J Campos H Hankinson SE Manson JE Stampfer MJ A prospective study of trans fatty acids in erythrocytes and risk of coronary heart disease Circulation 2007 115 1858 1865 10.1161/CIRCULATIONAHA.106.679985 17389261

[B12] Ascherio A Epidemiologic studies on dietary fats and coronary heart disease Am J Med 2002 113 9 9 12 10.1016/S0002-9343(01)00986-X 12566133

[B13] Kinsella JE Bruckner G Mai J Shimp J Metabolism of trans fatty acids with emphasis on the effects of trans, trans-octadecadienoate on lipid composition, essential fatty acid, and prostaglandins: an overview Am J Clin Nutr 1981 34 2307 2318 6794350 10.1093/ajcn/34.10.2307

[B14] Kummerow FA Zhou Q Mahfouz MM Smiricky MR Grieshop CM Schaeffer DJ Trans fatty acids in hydrogenated fat inhibited the synthesis of the polyunsaturated fatty acids in the phospholipid of arterial cells Life Sci 2004 74 2707 2723 10.1016/j.lfs.2003.10.013 15043986

[B15] Mozaffarian D Rimm EB King 1B Lawler RL McDonald GB Levy WC Trans fatty acids and systemic inflammation in heart failure Am J Clin Nuir 2004 80 1521 1525 10.1093/ajcn/80.6.1521 PMC1201402 15585763

[B16] De Roos NM Bots ML Katan MB Replacement of dietary saturated fatty acids by trans fatty acids lowers serum HDL cholesterol and impairs endothelial function in healthy men and women Arterioscler Thromb Vasc Biol 2001 21 1233 1237 10.1161/hq0701.092161 11451757

[B17] Harvey KA Arnold T Rasool T Antalis C Miller SJ Siddiquil RA Trans-fatty acids induce pro-inflammatory responses and endothelial cell dysfunction Br J Nutr 2008 99 723 731 17925051 10.1017/S0007114507842772

[B18] Maritim AC Sanders RA, Watkins 3rd JB. Diabetes, oxidative stress, and antioxidants: a review J Biochem Mol Toxicol 2003 17 24 38 10.1002/jbt.10058 12616644

[B19] Halliwell B Antioxidants and human disease: a general introduction Nutr Rev 1997 55 44 49 9155225 10.1111/j.1753-4887.1997.tb06100.x

[B20] Videla LA Rodrigo R Araya J Poniachik J Oxidative stress and depletion of hepatic longchain polyunsaturated fatty acids may contribute to nonalcoholic fatty liver disease Free Radic Biol Med 2004 37 1499 1507 10.1016/j.freeradbiomed.2004.06.033 15454290

[B21] Browning JD Horton JD Molecular mediators of hepatic steatosis and liver injury J Clin Invest 2004 114 147 152 15254578 10.1172/JCI22422 PMC449757

[B22] Videla LA Rodrigo R Araya J Ponichik J Insulin resistance and oxidative stress interdependency in non-alcoholic fatty liver disease Trends Mol Med 2006 12 555 558 10.1016/j.molmed.2006.10.001 17049925

[B23] Adams LA Angulo P Recent concepts in non-alcoholic fatty liver disease Diabetic Med 2005 22 1129 1133 10.1111/j.1464-5491.2005.01748.x 16108837

[B24] Safwat GM Pisanò S D'Amore E Borioni G Napolitano M Kamal AA Ballanti P Botham KM Bravo E Induction of non-alcoholic fatty liver disease and insulin resistance by feeding a high-fat diet in rats: does coenzyme Q monomethyl ether have a modulatory effect? Nutrition 2009 25 1157 1168 10.1016/j.nut.2009.02.009 19592219

[B25] Cano A Ciaffoni F Safwa GM Aspichueta P Ochoa B Bravo E Botham KM Hepatic very low density lipoprotein assembly is disturbed in a rat model of non alcoholic fatty liver disease: Is there a role for dietary Coenzyme Q? J Applied Physiol 2009 107 707 717 10.1152/japplphysiol.00297.2009 19608932

[B26] EEC (1991) Characteristics of olive oil and olive pomace and their analytical methods. Regulation EEC/2568/91 and latter modifications Off J Eur Commun 1991 248 1 82

[B27] Tura D Gigliotti C Pedo S Failla O Bassi D Serraiocco A Influence of cultivar and site of cultivation on levels of lipophilic and hydrophilic antioxidants in virgin olive oils (Olea europea L.) and correlations with oxidative stability Sci Hort 2007 112 108 119 10.1016/j.scienta.2006.12.036

[B28] Issaoui M Mechri B Echbili A Dabbou S Yangui AM Belguith H Trigui A Hammami M Chemometric characterization of five Tunisian varietals of olea europaea l. Olive fruit according to different maturation indices J Food Lipids 2008 15 322 328

[B29] Ramadan MF Morsel JT Radical scavenging activity of black cumin (Nigella sativa L.), coriander (Guizotia abyssinica cass,) crude seed oils and oil fractions J Agr Food Chem 2003 51 6961 6969 10.1021/jf0346713 14611155

[B30] Buege J Aust SD Colowick SP, Kaplan NO Microsomal Lipid Peroxidation Methods in Enzymology 1978 New York: Academic Press 302 311

[B31] Recknagel R Ghoshal AK Lipoperoxidation as a vector in carbon tetrachloride hepatotoxicity Lab Invest 1996 15 132 145 5951738

[B32] Beyer WE Fridovich I Assaying for superoxide dismutase activity: some large consequences of minor changes in conditions Anal Biochem 1987 161 559 66 10.1016/0003-2697(87)90489-1 3034103

[B33] Aebi H Catalase in vitro Methods Enzymol 1984 105 121 126 6727660 10.1016/s0076-6879(84)05016-3

[B34] Flohe L Gunzler WA Assays of glutathione peroxidase Methods Enzymol 1984 105 114 121 6727659 10.1016/s0076-6879(84)05015-1

[B35] Bradford MA rapid and sensitive for the quantification of microgram quantities of protein utilizing the principle of protein-dye binding Anal Biochem 1976 72 248 51 10.1016/0003-2697(76)90527-3 942051

[B36] Donnelly JK Robinson DS Free radicals in foods Free Radic Res 1995 22 47 76 10.3109/10715769509147527 7704185

[B37] Assumpção RP Dos Santos FD Andrade PMM Barreto GF do Carmo MGT Effect of Variation of Trans-Fatty Acid in Lactating Rats' Diet on Lipoprotein Lipase Activity in Mammary Gland, Liver, and Adipose Tissue Nutrition 2004 20 806 811 10.1016/j.nut.2004.05.004 15325692

[B38] Mayneris-Perxachs J Bondia-Pons I Moltó-Puigmartí C Mar Pairó AI Castellote M López-Sabater C Diet and plasma evaluation of the main isomers of conjugated linoleic acid and trans-fatty acids in a population sample from Mediterranean north-east Spain Food Chem 2010 123 296 305 10.1016/j.foodchem.2010.04.035

[B39] Lichtenstein AH Dietary trans fatty acid J Cardiopuhin Rehabil 2000 20 143 146 10.1097/00008483-200005000-00001 10860195

[B40] Kemneny Z Recseg K Henon G Kovari K Zwobada F Deodorization of vegetable oils: prediction of *trans *polyunsaturated fatty acid content J Ain Oil Chem Soc 2001 78 973 979 10.1007/s11746-001-0374-0

[B41] Uauy R Aro A Clarke R Ghafoorunissa R L'Abbé M Mozaffarian D Skeaff M Stender S Tavella M WHO Scientific Update on trans fatty acids: summary and conclusions Eur J Clin Nutr 2009 63 68 75

[B42] Varela SL Muniz FJS Cuest C Decreased food efficiency ratio, growth retardation and changes in liver fatty acid composition in rats consuming thermally oxidized and polymerized sunflower oil used for frying Food Chem Toxicol 1995 33 181 189 10.1016/0278-6915(94)00133-9 7896227

[B43] Ozaki M Fuchinoue S Teraoda S Ota K The in vivo cytoprotection of ascorbic acid against ischemia/reoxygenation injury of rat liver Arch Biochem Biophys 1995 318 439 445 10.1006/abbi.1995.1252 7733675

[B44] Edem DO Usoh IF Biochemical Changes in Wistar Rats on Oral Doses of Mistletoe (Loranthus micranthus) Am J Pharmacol Toxicol 2009 4 3 94 97

[B45] Mataix J Quiles JL Huertas JR Battino M Mañas M Tissue specific interactions of exercise, dietary fatty acids, and vitamin E in lipid peroxidation Free Radical Biol Med 1998 24 511 521 10.1016/S0891-5849(97)00288-8 9580480

[B46] Quiles JL Huertas JR Manas M Battino M Mataix J Physical exercise affects the lipid profile of mitochondrial membranes in rats fed with virgin olive oil or sunflower oil Br J Nutr 1999 81 21 24 10341671

[B47] Ramírez-Tortosa C Lopez-Pedrosa JM Suarez A Ros E Mataix J Gil A Olive oil-and fish oil-enriched diets modify plasma lipids and susceptibility of LDL to oxidative modification in free-living male patients with peripheral vascular disease: the Spanish Nutrition Study Br J Nutr 1999 82 31 39 10655954 10.1017/s0007114599001099

[B48] Yamaoka S Urade R Kito M Mitochondrial function in rats is affected by modification of membrane phospholipids with dietary sardine oil J Nutr 1988 118 290 296 3351630 10.1093/jn/118.3.290

[B49] Charnock JS McLennan PL Abeywardena MY Dietary modulation of lipid metabolism and mechanical performance of the heart Mol Cell Biochem 1992 116 19 25 10.1007/BF01270564 1480148

[B50] Quiles JL Huertas JR Battino M Ramırez-Tortosa MC Cassinello M Mataix J Lopez-Frias M Manňas M The intake of fried virgin olive or sunflower oils differentially induces oxidative stress in rat liver microsomes Br J Nutr 2002 88 57 65 12117428 10.1079/BJNBJN2002588

[B51] Nakbi A Tayeb W Grissa A Issaoui M Dabbou S Chargui I Ellouz M Miled A Hammami M Effects of olive oil and its fractions on oxidative stress and the liver's fatty acid composition in 2,4-Dichlorophenoxyacetic acid-treated rats Nutrition & Metabolism 2010 http://www.nutritionandmetabolism.com/content/7/1/80 21034436 10.1186/1743-7075-7-80 PMC2987329

[B52] Halliwell B Gutteridge JMC Free Radicals in Biology and Medicine 1999 3 Oxford University Press: Oxford, UK

[B53] Gutteridge JM Halliwell B Free radicals and antioxidants in the year 2000: a historical look to the future Ann N Y Acad Sci 2000 899 136 147 10863535 10.1111/j.1749-6632.2000.tb06182.x

[B54] Niu SL Mitchell DC Litman BJ Trans fatty acid derived phospholipids show increased membrane cholesterol and reduced receptor activation as compared to their cis analogs Biochem 2005 44 4458 4465 10.1021/bi048319+ PMC1779501 15766276

[B55] Brown DA Lipid rafts, detergent-resistant membranes, and raft targeting signals Physiol 2006 21 430 439 10.1152/physiol.00032.2006 17119156

[B56] Evans P Halliwell B Micronutrients: Oxidabt/antioxidant status Br J Nutr 2001 85 67 74 10.1079/BJN2000296 11509092

[B57] Thampi HBS Manoj G Leelamma S Menon VG Dietary fibre and lipid peroxidation: effects of dietary fibre on levels of lipids and lipid peroxides in high fat diet Ind J Exp Biol 1991 29 563 1653765

[B58] Batra S Singh SP Srivasta VML Xanthine oxidase, Superoxide dismutase, Catalase and lipid peroxidation in mastomys nataensis effect of dipentalonema viteae infection Indian J Exp Biol 1989 27 1067 2633968

[B59] Samuhasaneeto S Thong-Ngam D Kulaputana O Patumraj S Klaikeaw N Effects of N-Acetylcysteine on Oxidative Stress in Rats with Non-alcoholic Steatohepatitis J Med Assoc Thai 2007 90 4 17487136

[B60] Bradbury MW Berk PD Lipid metabolism in hepatic steatosis Clin Liver Dis 2004 8 639 671 10.1016/j.cld.2004.04.005 15331068

[B61] Robbins SL Pathologic basic of disease 1974 London: W. B. Saunders

[B62] Dashti N Feng Q Freeman MR Gandhi M Franklin FA Trans polyunsaturated fatty acids have more adverse effects than saturated fatty acids on the concentration and composition of lipoproteins secreted by human hepatoma HepG2 cells J Nutr 2002 132 2651 2659 12221225 10.1093/jn/132.9.2651

[B63] Mitmesser SH Carr TP Trans fatty acids alter the lipid composition and size of apoB-100 containing lipoproteins secreted by HepG2 cells J Nutr Biochem 2005 16 178 83 10.1016/j.jnutbio.2004.11.004 15741053

[B64] Brody T Nutritional biochemistry 1994 2 San Diego: Academic Press

[B65] Mensink RP Zock PL Kester AD Katan MB Effects of dietary fatty acids and carbohydrates on the ratio of serum total to HDL cholesterol and on serum lipids and apolipoproteins: a meta-analysis of 60 controlled trials Am J Clin Nutr 2003 77 1146 1155 12716665 10.1093/ajcn/77.5.1146

[B66] Bravo E Palleschi S Aspichueta P Buqué X Rossi B Cano A Mariarosaria N Ochoa B Botham KM High fat diet induced non alcoholic fatty liver disease in rats is associated with hyperhomocysteinemia caused by down regulation of the transsulphuration pathway Lipids Health Dis 2011 http://www.lipidworld.com/content/10/1/60 10.1186/1476-511X-10-60 PMC3096990 21504583

